# Variation of the essential oil components of *Citrus aurantium* leaves upon using different distillation techniques and evaluation of their antioxidant, antidiabetic, and neuroprotective effect against Alzheimer’s disease

**DOI:** 10.1186/s12906-024-04380-x

**Published:** 2024-02-02

**Authors:** Esraa A. Elhawary, Nilofar Nilofar, Gokhan Zengin, Omayma A. Eldahshan

**Affiliations:** 1 Department of Pharmacognosy, Faculty of Pharmacy, Ain Shams University, Abbassia, Cairo, 11566 Egypt; 2 Department of Biology, Science Faculty, Selcuk University, Campus, Konya Turkey; 3 Department of Pharmacy, Botanic Garden “Giardino dei Semplici”, Università degli Studi “Gabriele d’Annunzio”, via dei Vestini 31, Chieti, 66100 Italy; 4 Center for Drug Discovery Research and Development, Faculty of Pharmacy, Ain Shams University, Abbassia, Cairo, 11566 Egypt

**Keywords:** *Citrus aurantium*, Essential oil, Alzheimer’s, Microwave, Distillation

## Abstract

Citrus fruit essential oil is considered one of the widely studied essential oils while its leaves attract less attention although being rich in nearly the same composition as the peel and flowers. The leaves of bitter orange or sour orange (*Citrus aurantium* L.) were extracted using three different techniques namely; hydrodistillation (HD), steam distillation (SD), and microwave-assisted distillation (MV) to compare their chemical composition. The three essential oil samples were analyzed through GC/FID and GC/MS analyses. The samples were tested in vitro using different antioxidant techniques (DPPH, ABTS, CUPRAC, FRAP, PBD, and MCA), neuroprotective enzyme inhibitory activities (acetylcholine and butyl choline enzymes), and antidiabetic activities (α-amylase and α-glucosidase). The results showed that thirty-five volatile ingredients were detected and quantified. Monoterpenes represented the most abundant class in the three essential oils followed by sesquiterpenes. *C. aurantium* essential oil carried potential antioxidant activity where SD exhibited the highest antioxidant activity, with values arranged in the following order: FRAP (200.43 mg TE/g), CUPRAC (138.69 mg TE/g), ABTS (129.49 mg TE/g), and DPPH (51.67 mg TE/g). SD essential oil also presented the most potent α-amylase (0.32) inhibition while the MV essential oil showed the highest α-glucosidase inhibition (2.73 mmol ACAE/g), followed by HD (2.53 mmol ACAE/g), and SD (2.46 mmol ACAE/g). The SD essential oil exhibited the highest BChE and AChE inhibitory activities (3.73 and 2.06 mg GALAE/g), respectively). Thus, bitter orange essential oil can act as a potential source of potent antioxidant, antidiabetic, and neuroprotective activities for future drug leads.

**Supplementary Information:**

The online version contains supplementary material available at 10.1186/s12906-024-04380-x.

## Introduction

Essential oils and their components for a long time have been used in health, food, and aromatherapy. Essential oils are composed of many components, of varying concentrations and chemical structures [1, 2]. The growing interest in food safety is today gaining momentum for both the food industry and consumers as all are becoming more careful about the several health and environmental effects of foods. Natural products used in cosmetics and pharmaceutics processing today play a great role; their positive benefits on both health and the environment have made them the main principles of what formed a healthy diet today and, in the future [1, 2].

*Citrus* plant production has reached more than 126 million tons per year [3], where the Mediterranean region obsesses about one-fifth. This makes *Citrus* an economically important tree crop. *Citrus* is known to be rich in vitamins as B_9_, C and E, coumarins, dietary fiber and antioxidants [3–6].

The chemical composition of *Citrus* essential oil has been extensively studied and several compositional patterns owing to the species/cultivars, origin, climate, season, ripening stage, extraction, and analytical methods have been published. (*Citrus aurantium* L.) is a member of the family Rutaceae. It is famous in the Mediterranean region for many ornamental and agronomic uses. Peels and flowers represent the most-used parts of *C. aurantium* L. thus forming the base for many promising research groups due to their many medicinal properties [3]. *Citrus aurantium* L. var *amara* commonly named bitter or sour orange “Narenja Agria” [4], is widespread in Central and South America but mainly in the Mediterranean countries [5], where it is used in a large area from the industry as a flavoring agent or a fragrance to medicine as a nasal decongestant and a dieting agent [6]. The chemical compositions of leaves essential oils (LEO) and peel essential oils (PEO) from bitter oranges have been reported in the literature, where the main compounds in the LEO were linalool and linalyl acetate [7] whereas the PEO was dominated by limonene [7].

The chemical composition of the essential oil of sour orange (*Citrus aurantium*) was assessed in different plant parts during different seasons. Many studies were focused on the essential oil extracted from *C. aurantium* peels and limonene was found to be the major component. In contrast, other studies carried out on essential oil extracted from *C. aurantium* flowers showed that linalool and linalyl acetate are the main components. However, the volatile oil constituents from leaves have not received much attention in the literature. Indeed, the few reported studies that focused on *C. aurantium* leaves showed that linalool is the main component of essential oil. In addition, a large number of studies on *C. aurantium* were performed in Tunisia and Greece [3].

Sweet and bitter oranges are two of the most commercially important fruits with a huge world production due to their vast array of nutritive and therapeutic potentials. Sugar/acid ratios are regarded as the main index of citrus fruit maturity and one of the major analytical measures of its flavor quality [8]. Sweet orange plays an important therapeutic role in being active as an anti-diabetic, anti-obesity, and hypocholesterolemic agent. The peels and other waste by-products of the two kinds of citrus have a lot of therapeutic applications as nutraceuticals and functional foods. Peels and flowers, the most-used parts of *Citrus aurantium*, have constituted a largely promising area of research for their many medicinal properties. However, the leaves of sour orange have not yet been studied extensively. The essential oil of the bitter orange leaves from Algeria was analyzed using GC/MS. Forty-three volatile compounds were detected in essential oil and the majority of them were linalool, linalyl acetate, and *a*-Terpineol. The essential oil had an interesting level of elastase and collagenase inhibition and thus can be applied as an anti-aging agent [3, 9, 10]. This research article targeted the study of the effect of changing the essential oil extraction method on the composition of the essential oil of the leaves of *Citrus aurantium* together with the evaluation of its antioxidant, antidiabetic, and neuroprotective activities.

## Materials and methods

### Plant collection

The leaves of *Citrus* aurantium also known as bitter or sour orange were collected from a private farm in Shibin Al Kawm City, Al Minufiyah, Egypt in March 2023. The collected plant sample was authenticated by Dr. Usama K. Abdel Hameed, Department of Botany, Faculty of Science, Ain Shams University, Cairo, Egypt. A voucher specimen (voucher specimen number; PHG-P-CA-461) was deposited at the Department of Pharmacognosy, Faculty of Pharmacy, Ain Shams University, Cairo, Egypt.

### Essential oil preparation

#### Hydrodistillation

The dried rinds of *Citrus aurantium* (250 g) were subjected to four hours of hydrodistillation using a Clevenger-type glass apparatus. The oils were collected (1.4 ml) and then preserved at 4 °C in sealed vials for further analysis. The experiment was repeated three times.

#### Steam distillation

The dried rinds of *Citrus aurantium* (250 g) were subjected to three hours of steam distillation using a steam distillation apparatus. The oil (1.0 ml) in the organic layer was dried using anhydrous sodium sulfate and stored in a vial at room temperature for further analysis. The experiment was repeated three times.

#### Microwave-assisted hydro-distillation

A domestic microwave (MW), with an output power of 1000 Wattage, was assembled with a Clevenger apparatus. The dried *Citrus aurantium* (250 g) was homogenized and soaked in 500 mL distilled water transferred into the microwave and connected to the distillation apparatus. In the beginning, the MW was set at 50% of its power for 4 min (to initiate the boiling) then changed to 30% power for 40 min and left for 10 min to cool to yield 0.8 ml of the essential oil.

### GC/FID and GC/MS analysis

The GC/FID and GC/MS analysis were performed using the method adopted from [11].

### Antioxidant and enzyme inhibitory assays

To appraise the antioxidant potential of the extracts, six distinct spectrophotometric tests were conducted. This consisted of the ABTS and DPPH assays, that assess the antioxidants’ aptitude to neutralize free radicals. Through the FRAP and CUPRAC tests, the reduction potential of the extract was assessed. Phosphomolybdenum and ferrozine assays also measured the total antioxidant ability and metal chelating potential, respectively. Apart from MCA, each of these assays was evaluated with a Trolox standard. As for MCA, its comparison was made according to the EDTA equivalent per gram of essential oil. All procedures are detailed in our previous works [12, 13]. To investigate the inhibitory effects of the tested essential oils on various enzymes, we applied acetylcholinesterase (AChE), butyrylcholinesterase (BChE), tyrosinase, amylase, and glucosidase. Our earlier work provides information on the experimental procedures [12, 14]. We measured AChE and BChE inhibition in terms of milligrams of galanthamine equivalents (GALAE) per gram of essential oil, tyrosinase inhibition expressed as milligrams of kojic acid equivalents (KAE) per gram of essential oil, and α-amylase and α-glucosidase inhibition quantified as millimoles of acarbose equivalents (ACAE) per gram of essential oil. The details of experimental procedures are given in the supplementary materials.

### Statistical analysis

Antioxidant and enzyme inhibitory results were statistically evaluated. Statistical analysis was conducted with the help of Xl Stat (Version 16, Addinsoft Inc., New York, NY, USA). Results were expressed as mean ± standard deviation of 3 measurements. A significance level of *p* < 0.05 was applied to One-way ANOVA followed by Tukey’s post hoc test to identify statistically significant differences between groups.

## Results

### GC/MS analysis of *Citrus aurantium* leaves essential oil

**Fig. 1:**
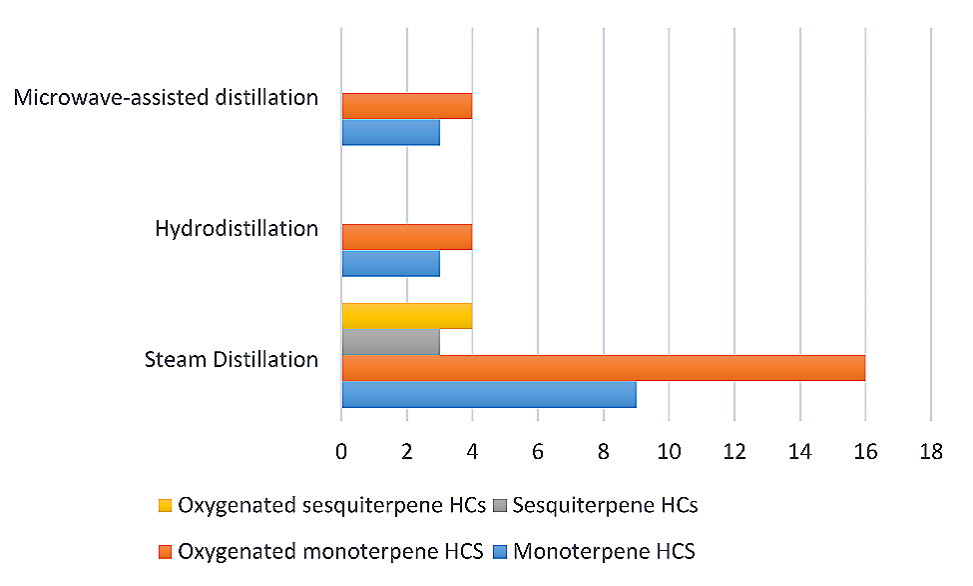
Bar chart showing the main identified classes of compounds from the three *C. aurantium* essential oils

The essential oil of the leaves of *Citrus aurantium* was prepared using three different distillation techniques namely; hydrodistillation (HD), steam distillation (SD), and microwave-assisted distillation (MV). The three prepared essential oils were analyzed using GC/FID and GC/MS analyses where thirty-five volatile components were qualitatively and quantitatively identified (Table 1). The % identification ranged from 99.97 to 99.98% with monoterpenes being the most abundant class in the three essential oils followed by sesquiterpenes (Fig. 1).

**Table 1:** Volatile components identified from *Citrus aurantium* leaves essential oil

No.	R_t_ (min.)	Component	KI			Molecular formula		% Composition		
			Calculated	Reported				Steam distilled (SD)	Hydrodistilled (HD)	Microwave-assisted (MV)
1.	6.904	β-Thujene	922	920		C_10_H_16_		0.21	0	0
2.	7.088	α-Pinene	929	931		C_10_H_16_		2.70	0	0
3.	7.505	Camphene	943	945		C_10_H_16_		0.11	1.32	0
4.	8.269	**4(10)-Thujene**	969	971		C_10_H_16_		**4.02**	0	0
5.	8.346	β-Pinene	971	977		C_10_H_16_		2.29	1.37	1.36
6.	8.766	2,3-Dehydro-1,8-cineole	986	986		C_10_H_16_O		0.24	0	0
7.	9.835	β-Myrcene	987	987		C_10_H_16_		0.15	1.33	1.29
8.	9.571	α-Terpinene	1012	1017		C_10_H_16_		0.14	0	0
9.	9.835	*p*-Cymene	1021	1025		C_10_H_14_		0.57	0	0
10.	10.063	**Eucalyptol**	1028	1031		C_10_H_18_O		**69.92**	0	0
11.	10.563	β-Ocimene	1044	1046		C_10_H_16_		0	0	1.38
12.	10.881	γ-Terpinene	1055	1056		C_10_H_16_		0.29	0	0
13.	11.123	*trans*-4-Thujanol	1062	1064		C_10_H_18_O		0.73	0	0
14.	12.155	**Linalool**	1096	1098		C_10_H_18_O		0.85	**24.04**	**23.69**
15.	13.345	*cis*-Pinocarveol	1135	1139		C_10_H_16_O		0.15	0	0
16.	14.076	*cis*-Dihydro-β-Terpineol	1159	1160		C_10_H_18_O		0.14	0	0
17.	14.221	6-Terpineol	1163	1166		C_10_H_18_O		0.29	0	0
18.	14.536	Terpinen-4-ol	1174	1177		C_10_H_18_O		1.52	0	0
19.	14.951	**α-Terpineol**	1187	1189		C_10_H_18_O		1.63	**5.14**	**7.82**
20.	15.123	(1R)-(-)-Myrtenal	1193	1192		C_10_H_14_O		0.25	0	0
21.	16.880	**Linalool acetate**	1253	1257		C_12_H_20_O		0	**64.08**	**57.13**
22.	17.753	Isobornyl acetate	1282	1288		C_12_H_20_O		0.18	0	0
23.	18.638	δ-Terpineol, acetate	1314	1316		C_12_H_20_O		0.43	0	0
24.	19.339	*cis*-2-acetoxy-1,8-cineole	1339	1341		C_12_H_20_O		0.22	0	0
25.	19.570	**α-Terpinyl acetate**	1347	1349		C_12_H_20_O		**9.86**	0	0
26.	19.819	3-Allylguaiacol; 3-Allyl-2-methoxyphenol	1356	1362		C_12_H_20_O		0.59	0	0
27.	19.925	**Geranyl acetate**	1360	1366		C_12_H_20_O_2_		0	2.70	**7.31**
28.	20.738	β-Elemene	1389	1389		C_15_H_24_		0.28	0	0
29.	21.079	Methyl eugenol	1402	1403		C_11_H_14_O		0.75	0	0
30.	21.503	Caryophyllene	1418	1420		C_15_H_24_		0.18	0	0
31.	23.279	α-Selinene	1486	1488		C_15_H_24_		0.11	0	0
32.	25.594	Spatulenol	1578	1578		C_15_H_24_O		0.20	0	0
33.	25.735	Caryophyllene oxide	1584	1583		C_15_H_24_O		0.46	0	0
34.	27.355	Eudesm-4(14)-en-11-ol	1652	1651		C_15_H_26_O		0.36	0	0
35.	34.860	Dehydrocostuslactone	2000	2006		C_15_H_20_O		0.15	0	0
		**% Composition**						**99.97%** **(32 cpd)**	**99.98%** **(7 cpd)**	**99.98%** **(7 cpd)**
		**Monoterpene HCS**						**9**	**3**	**3**
		**Oxygenated monoterpene HCS**						**16**	**4**	**4**
		**Sesquiterpene HCs**						**3**	**0**	**0**
		**Oxygenated sesquiterpene HCs**						**4**	**0**	**0**

*R_t_: retention time, HCs: hydrocarbons, KI: kovats index, cpd: compound

**Fig. 2:**
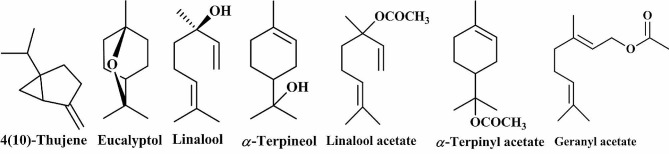
Structures of the major identified essential oil components from the three *C. aurantium* essential oils

Eucalyptol (69.92%), α-terpinyl acetate (9.86%), and 4 (10)-thujene (4.02%) were the major identified from the steam distilled essential oil while the hydrodistilled *C. aurantium* essential oil showed the presence of linalool acetate (64.08%), linalool (24.04%) and α-terpineol (5.14%) as the main identified components. Moreover, linalool acetate (57.13%), linalool (23.69%), α-terpineol (7.82%), and geranyl acetate (7.31%) were the main ingredients of the microwave-assisted isolated essential oil (Table 1; Fig. 2).

As mentioned earlier the monoterpene class of compounds were among the main identified class with its two types, the non-oxygenated and the oxygenated types. Eucalyptol (69.92%), linalool acetate (23.69-64.08%), linalool (23.69-24.04%), α-terpineol (5.14-7.82%), geranyl acetate (7.31%) and 4(10)-thujene (4.02%). In addition to that, sesquiterpenes were the second most abundant class and were only traced from the steam distilled essential oil with β-elemene (0.28%), caryophyllene (0.18%) and α-selinene (0.11%) as the main identified group from the sesquiterpene class while caryophyllene oxide (0.46%), eudesm-4(14)-en-11-ol (0.36%), spatulenol (0.20%) and dehydrocostuslactone (0.15%) were the major components from the oxygenated sesquiterpenes.

### Study of the biological activities of citrus aurantium leaves essential oil

#### Antioxidant activity

A wide array of assays can be employed to directly measure the transfer of hydrogen atoms or electrons from potential antioxidants to free radicals in simplified “lipid-free” systems. The reported antioxidant activities obtained through these methods are typically linked to their ability to scavenge specific types of radical species, some of which may be artificially generated and biologically insignificant [15]. Consequently, many antioxidant assays are employed to assess the antioxidant potential of natural products. In the current study, six distinct antioxidant assays were utilized namely, DPPH, ABTS, CUPRAC, FRAP, PBD, and MCA. The results of the antioxidant assays indicated that the essential oil extracted by SD exhibited the highest antioxidant activity, with values arranged in the following order: FRAP (200.43 mg TE/g), CUPRAC (138.69 mg TE/g), ABTS (129.49 mg TE/g), and DPPH (51.67 mg TE/g). In the case of the MV-extracted essential oil, the order of antioxidant activity is consistent with that of the SD extract, with values arranged as follows: MV FRAP (57.21 mg TE/g), CUPRAC (37.44 mg TE/g), ABTS (35.75 mg TE/g), and DPPH (15.00 mg TE/g). Moreover, the MV extraction method essential oil demonstrated the highest PBD (37.87 mmol TE/g) and MCA (32.14 mmol TE/g) values. Conversely, the HD-extracted essential oil exhibited comparatively lower activity in all the antioxidant tests, particularly in the case of DPPH, where it recorded only 10.41 mg TE/g (Table 2).

**Table 2:** Antioxidant effects of the three *Citrus aurantium* essential oil samples

Methods	DPPH (mg TE/g)	ABTS (mg TE/g)	CUPRAC (mg TE/g)	FRAP (mg TE/g)	PBD (mmol TE/g)	MCA (mg EDTAE/g)
HD	10.41 ± 1.27^c^	11.05 ± 0.50^c^	16.15 ± 0.32^c^	29.43 ± 0.29^c^	18.60 ± 0.04^c^	20.44 ± 0.89^c^
SD	51.67 ± 0.69^a^	129.49 ± 0.29^a^	138.69 ± 0.27^a^	200.43 ± 2.88^a^	24.03 ± 0.09^b^	22.49 ± 0.80^b^
MV	15.00 ± 0.42^b^	35.75 ± 0.32^b^	37.44 ± 1.05^b^	57.21 ± 0.52^b^	37.87 ± 0.06^a^	32.14 ± 0.19^a^

*Values are reported as mean ± SD of three parallel experiments. TE: Trolox equivalent; EDTAE: EDTA equivalent. Different letters (^a−c^) indicate significant differences in the tested essential oils

#### Enzymes inhibitory activity

Compounds that influence the rates of enzyme-catalyzed reactions are called modulators. Those whose effect is to reduce the rate is called inhibition [16]. Enzyme inhibitors have diverse applications, including pharmaceuticals for humans and animals, as well as herbicides and pesticides. The process of enzyme inhibition is a subject of extensive examination due to its significant relevance in understanding enzyme mechanisms [17] and its role in pharmacological research [18, 19]. As an illustration, Alzheimer’s disease (AD), a major neurodegenerative disorder, predominantly affects the older and pre-elderly population [20]. This neurological disorder results from a shortage of acetylcholine (ACh) and is characterized by heightened brain tissue degeneration. The enzymes acetylcholine esterase (AChE) and butylcholine esterase (BChE) are identified as the key players in the hydrolysis of ACh in the human body [21].

Table 3 indicates that the SD-extracted essential oil exhibited the highest BChE inhibition activity, with a measurement of 3.73 mg GALAE/g, and AChE inhibition, with a value of 2.06 mg GALAE/g. Following closely, the HB-extracted essential oil demonstrated BChE inhibition at 2.92 mg GALAE/g and AChE inhibition at 2.00 mg GALAE/g. In contrast, the MV-extracted essential oil displayed the lowest activity, with 0.66 mg GALAE/g for AChE inhibition and 0.39 mg GALAE/g for BChE inhibition. These findings align with the antioxidant activity observed in the extracted oil, as presented in Table 2.

Tyrosinase plays a pivotal role in initiating the synthesis of melanin, leading to the browning phenomena observed in fruits, vegetables, and mushrooms upon bruising or prolonged storage. In the realm of mammals, tyrosinase not only contributes to the pigmentation of hair and skin [22], but it also influences skin conditions, giving rise to hypo (such as vitiligo) or hyper (manifesting as flecks or freckles) pigmentation [23]. This has led to a notable upsurge of interest in researching, developing, and screening potent tyrosinase inhibitors, making it a prominent focus in agriculture, food, medicine, and cosmetics [24]. Table 3 demonstrates that the essential oil extracted from HD exhibited a significantly higher inhibition activity of 62.51 mg KAE/g, whereas the essential oil from SD exhibited an inhibition rate of 44.62 mg KAE/g. Notably, it was observed that the essential oil extracted from MV did not exhibit any significant activity against tyrosinase.

Type 2 *Diabetes mellitus* (T_2_DM) is a common health issue with elevated blood glucose levels that can lead to severe complications, including nephropathy, neuropathy, and cardiovascular disease [25]. In the management of T_2_DM, the inhibition of pancreatic α-amylase and α-glucosidase enzymes, responsible for breaking down dietary carbohydrates, can result in delayed glucose uptake, reduced carbohydrate digestion, and a decrease in blood sugar levels [26]. This approach is crucial in controlling postprandial hyperglycemia in T_2_DM [26, 27]. The current research findings reveal that SD essential oil exhibited the greatest α-amylase inhibition at 0.32, surpassing the HD essential oil, which measured inhibition at 0.19. Regarding α-glucosidase inhibition, MV essential oil demonstrated the highest level of α-glucosidase (2.73 mmol ACAE/g), followed by HD (2.53 mmol ACAE/g), and SD (2.46 mmol ACAE/g).

**Table 3:** Enzyme inhibitory effects of the three *Citrus aurantium* essential oil samples

Methods	AChE (mg GALAE/g)	BChE (mg GALAE/g)	Tyrosinase (mg KAE/g)	α-Amylase (mmol ACAE/g)	α-Glucosidase (mmol ACAE/g)
HD	2.00 ± 0.02^a^	2.92 ± 0.08^b^	62.51 ± 0.94^a^	0.19 ± 0.03^b^	2.53 ± 0.03^b^
SD	2.06 ± 0.04^a^	3.73 ± 0.06^a^	44.62 ± 6.74^b^	0.32 ± 0.01^a^	2.46 ± 0.01^c^
MV	0.66 ± 0.05^b^	0.39 ± 0.07^c^	na	0.12 ± 0.02^c^	2.73 ± 0.01^a^

*Values are reported as mean ± SD of three parallel experiments. GALAE: Galanthamine equivalent; KAE: Kojic acid equivalent; ACAE: Acarbose equivalent; na: not active. Different letters (^a−c^) indicate significant differences in the tested essential oils

## Discussion

Plant extracts and essential oils have gained much interest recently due to their interesting chemical composition together with potent biological activities which positioned them on top of research interests worldwide [28–30]. Genus *citrus* is famous for its well-known species viz. orange, lemon, lime, grapefruit, and many others. *Citrus* essential oils were documented to contain many oxygenated and non-oxygenated terpenes. The volatile components were usually isolated from the leaves [3, 31], flowers [32], fruits [33], fruit rind [34], bark [35], and even the seeds [36]. Many biological activities were reported for the *Citrus* essential oils including; antiviral [29, 37], antibacterial [28, 38, 39], anti-inflammatory [40, 41], antioxidant [38, 42], cytotoxic [43–46], etc.

Sour orange also known as *Citrus aurantium* is one of the famous and well-studied *Citrus* species, especially its essential oil content. Herein, one hundred and two volatile components were identified and quantified from three essential oil samples of *C. aurantium* and they were different in the oil preparation method. As discussed earlier in the results, the hydrodistilled and microwave-assisted extraction of the sour orange essential oils showed nearly similar volatile oil contents with the same components in both of them with differences in the compositions of these components. Moreover, the hydrodistilled essential oil (HD) showed the unique presence of camphene while the microwave-assisted (MV) showed the presence of β-ocimene which was absent in the HD essential oil. On the other hand, the steam-distilled (SD) essential oil presented a unique chemical composition when compared to the two other oil samples. β-Pinene, β-myrcene, linalool, and α-terpineol were found to be common components for the three essential oil samples while the latter two components were major in the HD and MV essential oils they represented minor or even trace components of the SD essential oil.

As reported in the literature, steam distillation marks the most recommended method for extraction of *Citrus* essential oils compared to other conventional methods [47] due to its ability to allow the liberation of the volatile components under controlled conditions of pressure and temperature compared to the atmospheric pressure in case of hydrodistillation for example [48, 49]. Other studies on *C. aurantium* essential oil addressed the effect of the isolation technique on both the quality and the essential oil yield. One study compared the essential oil components from *C. aurantium* blossoms obtained through seven isolation methodologies namely; commercial hydrodistillation, hydrodistillation, steam distillation, ohmic-assisted hydro distillation, solvent-less microwave extraction, solvent-free microwave extraction and microwave-assisted hydrodistillation where hydrodistillation showed the highest essential oil yield compared to the other methods [50].

Another study compared four isolation techniques namely; hydrodistillation, solvent extraction, microwave-assisted extraction, and ultrasound-assisted extraction regarding the essential oil composition of the peels of *C. aurantifolia, C. limon* and *C. sinensis.* This study concluded that both hydrodistillation and solvent extraction were superior in the isolation of mono- and sesquiterpenes while the two other methods led to the isolation of essential oils with higher percentages of hydrocarbons, oxygenated monoterpenes, sesquiterpenes, oxygenated sesquiterpenes and fatty acids [51]. Thus, upon the results of such studies and when comparing them to our current study. The essential oil composition can vary according to many factors mainly centered on the isolation technique, the part used in the extraction process, the different species also the seasonal variation. All or part of these factors eventually led to new essential oil components even from the same plant species thus proper selection and optimization of the distillation technique plays crucial role in the quality and quantity of the resulting essential oil.

Many earlier studies had highlighted the chemical components and medical importance of sour orange essential oils. The essential oil from *C. aurantium* peels was rich with limonene as the main component with about 90% of the total oil content and it exhibited potent antioxidant activities with ABTS^•+^ (44.93 ± 1.45%) and DPPH^•^ (11.03 ± 1.08%) inhibition [52]. The essential oil of *C. aurantium* leaves was evaluated for its composition together with its potential antioxidant and anti-inflammatory activities. The essential oil contained forty-three components (yield = 0.57%). Linalool, linalyl acetate, and α-terpineol composed the bulk of the essential oil composition. The antioxidant activity was evaluated through a DPPH assay (IC_50_ > 10,000 mg/L) [3].

The hydrodistilled essential oil contents of the leaves and peel of *C. aurantium* were compared where the leaves essential oil contained linalool (18.6%), γ-terpinene (6.9%) and α-terpineol (15.1%) as its main compounds while the peel essential oil was rich in linalool (12%), *cis-*linalool oxide (8.1%), *trans*-carveol (11.9%), *endo*-fenchyl acetate (5.5%) and carvone (5.8%) [7].

Different herbal extracts and essential oils represent promising reservoirs of naturally occurring compounds that exhibit antioxidant properties. A prior investigation has documented the most robust DPPH scavenging activity within the *C*. *aurantium* extract, demonstrating IC_50_ values of 96.07 µg/ml, and its corresponding essential oil, with IC_50_ values of 393.71 µg/ml [53]. The essential oil extracted through SD exhibited elevated concentrations of both monoterpene and sesquiterpene components. A prior investigation showed their antioxidant effectiveness, which closely paralleled that of the phenolic constituents found in *C*. *aurantium* [53]. These molecules effectively disrupted free-radical chain reactions and induced their transformation into inert compounds, as supported by additional studies [54, 55]. The findings of our study align with those of several other researchers who have affirmed the antioxidant capabilities of *Citrus* essential oils [3, 53, 54, 56]. Nonetheless, presenting their antioxidant efficacy in IC_50_ values makes it challenging to make a direct comparison with the current study, given the difference in units of measurement.

The current study assessed the therapeutic potential of essential oil extracted from *C. aurantium* using three distinct extraction methods in the context of AD treatment. This evaluation involves the examination of their effects on AChE and BChE inhibition assays. In AD pharmacotherapy, AChE and BChE inhibitors are frequently used to target essential enzymes, easing cognitive symptoms and slowing disease progression [25]. The elevated monoterpenes content in Citrus essential oils provides a plausible rationale for their bioactivity. Numerous studies have underscored the AChE inhibitory properties associated with this category of secondary metabolites. Previous research has explored the potential of various monoterpenoids from *Citrus* essential oils, including monoterpene, oxygenated monoterpene, and sesquiterpene compounds, for inhibiting cholinesterase enzymes, with a significant focus on AChE inhibition [57, 58]. There are reports of certain citrus essential oils displaying radical scavenging capabilities [59]. For instance, Eureka lemon essential oil, known for its potent tyrosinase inhibitory properties, has also been observed to exhibit DPPH radical scavenging activity [60]. Previous study and the present results suggests that citrus essential oils, composed of a diverse array of compounds, may effectively inhibit melanogenesis through a multifaceted range of mechanisms and activities.

To manage diabetes, reducing post-meal high blood sugar levels involves inhibiting two crucial digestive enzymes: α-amylase and α-glucosidase, responsible for breaking down complex carbohydrates into simpler sugars. This study unveiled the potential antidiabetic properties of *C. aurantium* essential oil by demonstrating their inhibition of α-amylase and α-glucosidase in vitro, corroborating prior citrus extract research [61, 62]. These findings, along with previous studies, suggest that the presence of monoterpenes and sesquiterpenes, along with their synergistic interactions, likely underlie these enzymes’ suppressive effects of the essential oils.

The observed enzyme inhibitory effects can be explained by the presence of some volatile components in the tested essential oils. For example, the SD essential oil was rich in eucalyptol and the compound had a potent cholinesterase inhibition [63–65], amylase inhibition [66] and glucosidase inhibition [67]. Regarding the HD and MW essential oils contained higher concentration of linalool and linalool acetate. Similarly, some studies have been found significant enzyme inhibitory properties of the compounds; Acetylcholinesterase; [68, 69], tyrosinase [70], glucosidase inhibition [71]. However, as can be seen in Table 1, the essential oils contained several compounds, so their synergistic and antagonistic effects are possible. In this sense, we proposed further studies to isolate the components and test their enzyme inhibitory potential as individuals.

In the current study, components like linalool acetate, α-terpinyl acetate, linalool, and eucalyptol represented the main essential oil components obtained through the three studied distillation techniques. The aforementioned components belong to the oxygenated monoterpene class of volatile constituents which is well-known for its potent antioxidant and neuroprotective effects through their ability to fight against oxidative stress and support the natural oxidant-antioxidant balance [72–74] as well as their potential antidiabetic activity through inhibition of α-amylase and α-glucosidase as the main enzymes involved in the process [75]. Moreover, they can modulate enzymes and proteins that contribute to insulin resistance and other pathological events caused by *Diabetes milletus* [76].

## Conclusion

It can be concluded that the essential oil of *Citrus aurantium* represented a valuable source of essential oil components viz. linalool acetate, α-terpinyl acetate, linalool and eucalyptol. The different applied extraction methods in this article namely, hydrodistillation, steam distillation and microwave-assisted distillation provided different patterns of volatile constituent distribution although steam distillation was somehow different from the two other methods in its components. Monoterpenes were the main identified compounds followed by sesquiterpenes. The three sour orange essential oils showed promising antioxidant, antidiabetic, and neuroprotective activities in vitro through enzyme inhibition. The postulated biological effects were attributed to the presence of a higher percentage of oxygenated monoterpenes in the three essential oil samples. The authors recommend future *in silico* and in vivo studies on the main identified essential oil constituents against the potential biological binding sockets also further analysis of the chemical composition of the *Citrus aurantium* leaf essential oil using more advanced extraction techniques will give a wider view of the effect of varying the distillation process on the essential oil components which will further affect the biological activity.

### Electronic supplementary material

Below is the link to the electronic supplementary material.

Supplementary Material 1

## Data Availability

Data are available upon request from the first author, Esraa A. Elhawary, esraa.elhawary@pharma.asu.edu.eg.
